# Methodology of Leakage Prediction in Gasketed Flange Joints at Pipeline Deformations

**DOI:** 10.3390/ma15124354

**Published:** 2022-06-20

**Authors:** Przemysław Jaszak, Janusz Skrzypacz, Andrzej Borawski, Rafał Grzejda

**Affiliations:** 1 Faculty of Mechanical and Power Engineering, Wroclaw University of Science Technology, 27 Wybrzeze Stanislawa Wyspianskiego Str., 50-370 Wroclaw, Poland; janusz.skrzypacz@pwr.edu.pl; 2 Faculty of Mechanical Engineering, Bialystok University of Technology, 45C Wiejska Str., 15-351 Bialystok, Poland; a.borawski@pb.edu.pl; 3 Faculty of Mechanical Engineering and Mechatronics, West Pomeranian University of Technology in Szczecin, 19 Piastow Ave., 70-310 Szczecin, Poland; rafal.grzejda@zut.edu.pl

**Keywords:** gasketed flange joint, Finite Element Method, gasket, design by analysis

## Abstract

The paper presents the proposal of a leakage prediction method in flange joints, after pipeline deformation, based on FEM (Finite Element Methods). The stages of developing the design are discussed, and a complex, multi-stage method of applying the loads is presented in detail. Moreover, the gasket material data obtained in experiments were used. The paper also presents the results of calculations on a non-uniform stress distribution in the radial direction of the gasket. In addition, it has been shown that the deflection of the pipeline with a minor displacement causes an increase in the diversification of the circumferential pressure of the gasket, and also has a significant influence on the determination of the actual state of stress to which the gasket is subject. Moreover, it was found that the distribution of contact pressure on the deflection of the pipeline has a significant influence on the level of leakage. The results of tests are compared to the results of the numerical calculations of the stress in bolts. By comparing the bolt tension changes obtained by numerical and experiment analyses, it has been shown that the assumptions made in developing the numerical model are correct.

## 1. Introduction

Tightness is one of the most important design criteria for almost any industrial installation. Elements of installations are connected to each other in a non-detachable (most often welded joints) or detachable (most often flanged joints) manner with the use of various types of gaskets [1,2,3]. In order for a flange joint to remain tight, the pressure on a gasket—in any area of its surface—must not fall below a certain minimum value, which ensures no leakage. When designing a flange joint, it is mainly assumed that it is tensed by forces that originate from internal pressure. Nevertheless, the deformation of the entire pipeline also affects the state of stresses and deformation between the elements of such joints [4].

For complex stress and deformation conditions in machine elements, the DBA (Design by Analysis) method has become more and more popular. However, the FEM (Finite Element Method) is most commonly used as the analysis tool. A gasketed flange joint is a structure with a complex state of stresses. Moreover, sealing material exhibits a very high non-linearity and plasticity. For this reason, the use of the DBA method is absolutely justified in this case.

The concept of using FEM for the analysis of flange joints is not new [3,5]. The authors of paper [6] analysed the impact of mesh compaction within the contact surface area of a gasket with a flange-raised face in order to check the accuracy of the numerical solution. Papers [7,8] considered the impact of the non-linear characteristics of the gasket material on the bolt load condition. It was discovered that the tension was distributed more uniformly in joints with more rigid gaskets. Many papers have investigated the impact of various contact issues on the accuracy of the numerical solution [9,10,11]. Moreover, studies [12,13] analysed the influence of the rigidity of a gasket on the bolts’ tension level caused by the increase in pressure of the sealed medium. However, these models did not take into account the non-linear characteristics of gaskets. Paper [14] presents a method of numerical modelling of leakage from a flange joint subject to an operating load and considers the impact of elevated temperature on the deformation of the joint elements. Paper [5] describes a non-linear method of modelling a flanged-bolted joint operating under elevated temperature conditions. The following issues were analysed using the Transient Thermal Analysis method: stress relaxation in the bolts, deformation of the flanges as a result of thermal expansion of material, and the loss of contact on the contact surface of raised faces. Papers [15,16] present the modelling of a planar model of a thermomechanically loaded bolted joint.

Based on a review of the literature concerning bolted-flange joints with a gasket, it can be concluded that there is no comprehensive approach to the modelling of this structure with consideration of the pipeline deflection and its impact on the leakage level.

This paper is devoted to the assessment of the stress condition of static seals (gaskets) working in flanged-bolted joints. It is common knowledge that a number of factors influence the tightness of flange joints, e.g., the kind of applied gasket (i.e., its rigidity), the in-service tension of bolts, sealed fluid pressure, the external load caused by the pipeline weight, or (static and/or) pipeline bending caused by the external load. Under such complex loading conditions, the distribution of contact pressure acting on the gasket is diversified. The prerequisite to ensure the tightness of flange joints is to maintain the correct value of residual pressure on the gasket, which in turn ensures appropriate tightness [17,18,19]. In order to identify the residual pressure distribution on the gasket (which results from the complex load condition of a flange joint), a numerical model of such a joint was constructed. The geometrical dimensions of individual elements of this model corresponded to the elements that were used in the experimental tests. The same operating conditions as those used for the experimental tests were modelled in the numerical analyses.

The aim of the research is to verify to what extent the bending of a pipeline affects the tightness of a flanged-bolted joint. In the first part of the work, numerical calculations were performed, in which it was analysed to what extent the spacing between the pipeline’s supports affects the deflection value and the load condition of the flange-bolted joint. For this purpose, the CAEPIPE calculation program was used, which is based on the EN 13480 calculation code [20]. An industrial pipeline with dimensions corresponding to the PN100 DN63 designation was adopted for the analysis. In the second part of the work, experimental tests were carried out, in which the influence of the pipeline’s deflection on the leakage level and the load condition of bolts was assessed. For this purpose, a laboratory stand was built. It consisted of a fragment of industrial pipeline DN100 PN63 with a flanged-bolted joint and a soft material gasket. In the third part of the work, numerical calculations that simulated the laboratory load conditions of the pipeline were carried out. The purpose of these calculations was to determine the pressure distribution on the surface of the gasket, which occurred during the complex load condition of the pipeline. These calculations enabled the average pressure value and its correlation with the leakage level to be determined.

## 2. Materials and Methods

**Figure 1:**
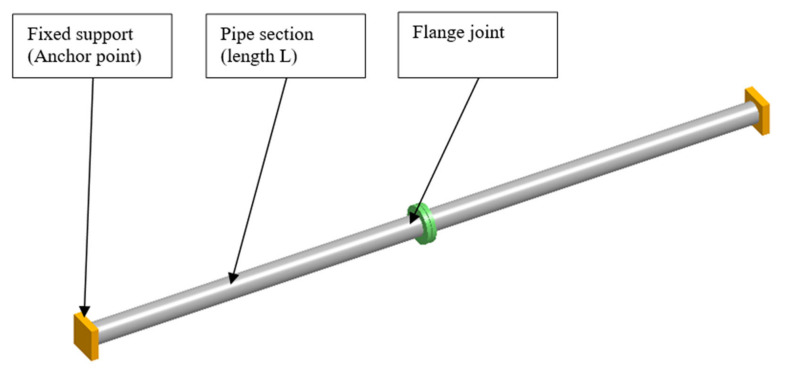
Model of the pipeline.

The object of this research is the flange joint that connects two parts of a pipeline. The pipeline is supported by two fixed supports located at the ends (Figure 1).

**Figure 2:**
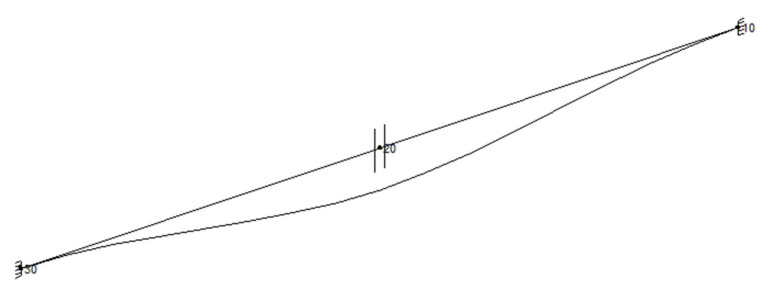
Example of graphical results: pipeline deflection for *L* = 1850 mm.

**Figure 3:**
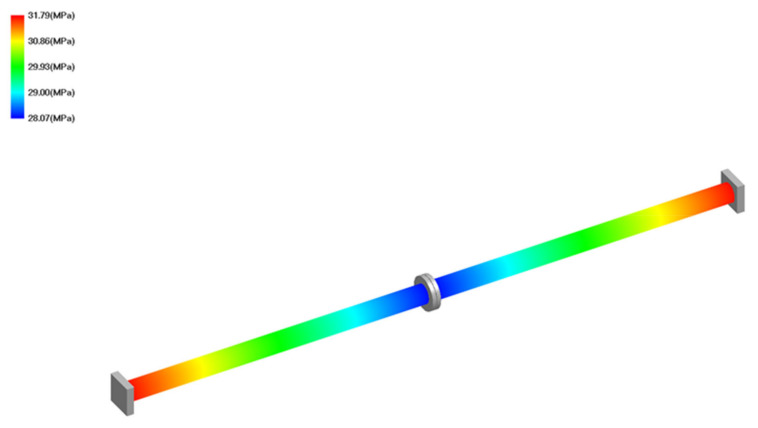
Example of graphical results: sustained stress *σ*_1_ (MPa) for L = 1880 mm.

The geometrical and operating parameters of the model are presented in Table 1. The material for flanges, pipes, and supports was austenitic stainless steel 304 (1.4301). The three pipe section lengths *L* were taken into account: 1850 mm, 2250 mm, and 2500 mm. The maximal value of length *L* was determined in accordance with the rule that the maximal distance between supports is about 5000 mm for pipe DN100 and pressure PN25. In order to determine the pipe’s displacement in the location of the flange joint, flexibility analysis was performed using CAEPIPE software. Results of the calculations are presented in Table 2, and examples of graphical results are shown in Figure 2 and Figure 3. The geometrical model (Figure 1) represents the following elements of a flange joint: pipelines with flanges, pipeline supports, gasket, and bolts.

### 2.1. Experimental Method

**Figure 4:**
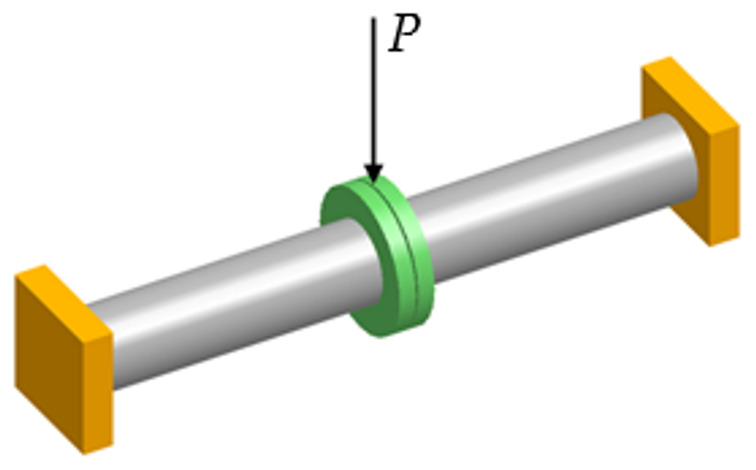
Model for the force value calculations.

**Figure 5:**
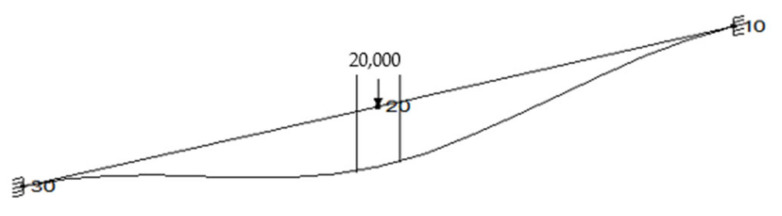
Example of the graphical results of the shorter model: pipeline deflection for *δ =* 0.4 mm.

In order to examine the influence of gas pressure and joint bending on the leakage level, a special test rig was designed and built. In the experimental test, the pipe was shortened to 1000 mm. The reason for this was the limited laboratory space as well as the maximal force generated by the hydraulic piston that bent the pipeline. In order to maintain the same displacement in the flange joint location, an additional radial force had to be applied. The value of such a force was determined by means of a flexibility analysis with the CAEPIPE software (SST Systems, Inc., San Jose, CA, USA), according to Figure 4. Example of the graphical results of the shorter model is shown in Figure 5.

Results of the calculation are as follows:

- Displacement *δ* = 0.4 mm, force *P* = 20 kN;
- Displacement *δ* = 0.8 mm, force *P* = 70 kN;
- Displacement *δ* = 1.2 mm, force *P* = 120 kN.

**Figure 6:**
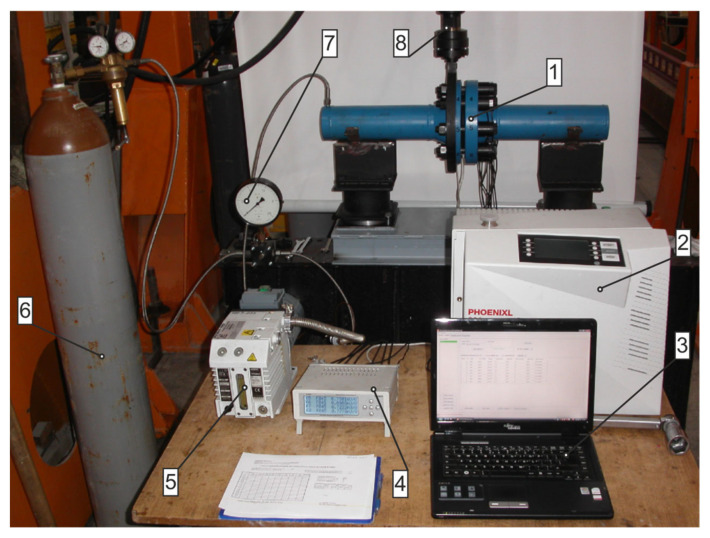
Test rig: 1—tested flanged-bolted joint; 2—helium detector; 3—PC recording the change in bolt tension; 4—strain-gauge amplifier; 5—vacuum pump; 6—cylinder with helium; 7—pressure gauge; 8—head connecting the hydraulic actuator with the tested object.

Figure 6 shows the stand where the experimental tests were carried out. The stand was used, e.g., for evaluation of the tightness of static gaskets operating under forced vibrations. A flanged-bolted joint (1) of two sections of pipes plugged with bottom plugs at both ends makes up the basic element of the stand. The construction, after mounting the gasket between the connected surfaces of the flanges, and appropriate tensioning of the joint bolts make a closed pressure vessel. The dimensions of the flanges complied with the PN-EN 13445-1 standard [21] (according to Table 1), with raised faces of type B and sealed fluid pressure of PN 100 bar. The tank was loaded with a pressure (2 MPa) of gas supplied from the reservoir (6). The model pipeline fragment rested on two supports: a right fixing support and a left displacement support. In order to induce variable pipeline loads (bending), the left flange was tightly fitted in a metal ring, which was then connected with the head (8), in turn making a part of the hydraulic cylinder act as a forced generator.

In order to identify the value of the bolt’s tension, TFpxy-4/350-type foil strain gauges were placed on each bolt and then connected to form a full bridge set-up. The value of the voltage signal of the strain-gauge bridge was amplified in an eight-channel static signal amplifier (4). The static-signal amplifier was connected to the computer (3). The gas leaking from the joint was measured with a spectrometric helium detector, “Phoenikl 300” (2) (Leybold GmbH, Köln, Germany), equipped with a vacuum pump (5).

After mounting the seal between the flanges, the joint was bolted. The bolts were tightened with a torque wrench in three steps, which represented 30, 60, and 100% of the target value. The target value was set at 52 kN (per bolt), and it was read directly from the strain gauge signal recorder. Twenty bar helium was applied to the joint and the leakage level was then measured. The joint was then subjected to bending of 0.4 mm, 0.8 mm, and 1.2 mm. Leaking helium and the bolt tension force were measured at each deflection point.

### 2.2. Numerical Method

**Figure 7:**
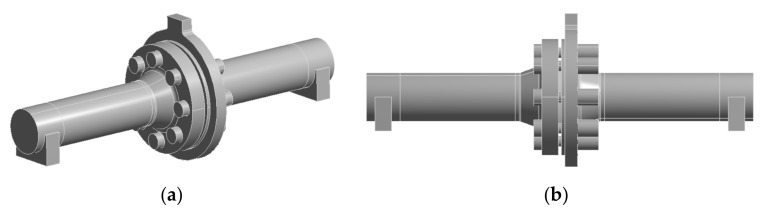
CAD model of the considered flanged-bolted joint of the pipeline fragment: (**a**) ISO view, (**b**) view from the side.

Numerical methods were used to better investigate the problem of pipeline deflection leading, in general, to uneven tension in bolts and also to an increase in the leakage. The geometrical model used in the numerical calculations is shown in Figure 7.

The calculating model was prepared in ANSYS Workbench 15.0 software (ANSYS, Inc., Canonsburg, PA, USA) with the use of static structural analysis. A computational mesh was generated and mainly based on the “HEXA” type of elements. The discretization of gaskets was carried out with the use of dedicated “GASKET”-type elements, which are intended only for static gasket modelling.

**Figure 8:**
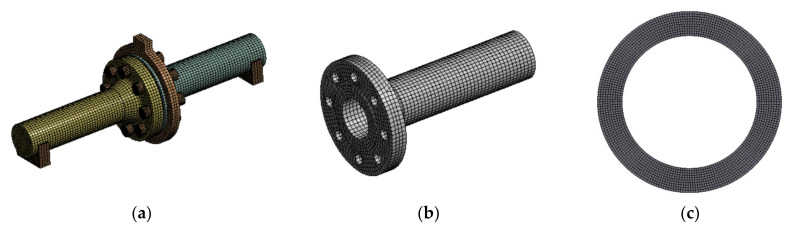

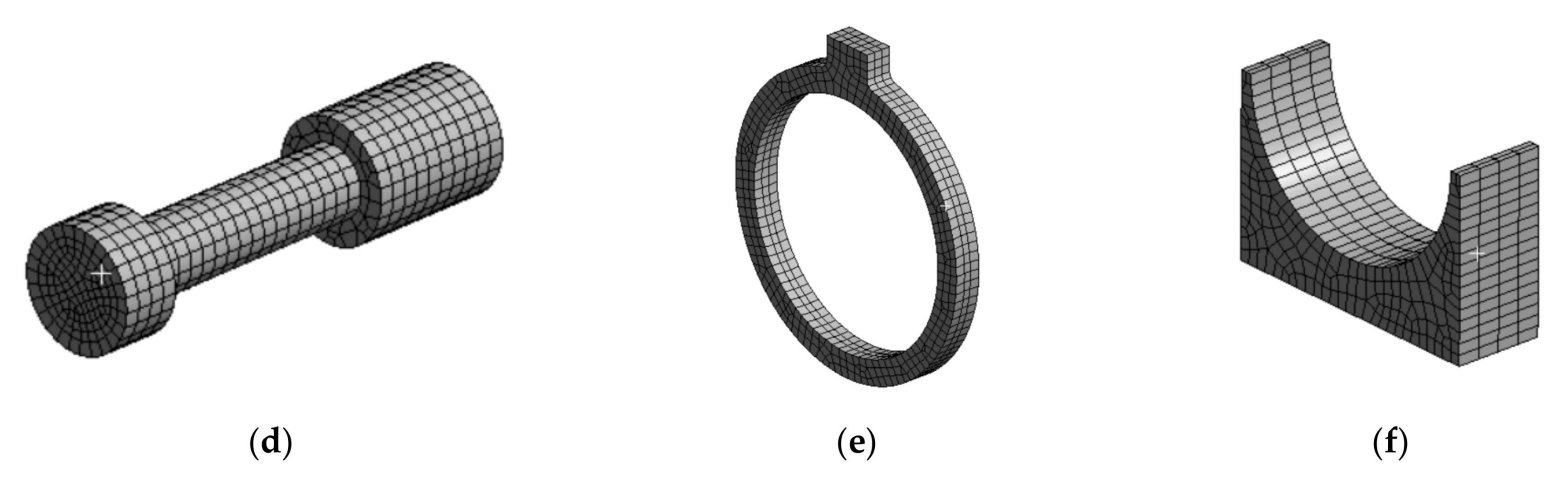
Discretization of individual elements of the analysed pipeline fragment: (**a**) assembly; (**b**) flange; (**c**) gasket; (**d**) bolt; (**e**) ring; (**f**) support.

Mapping of the contact surface was used in the contact places between the gasket and the flange-raised face. The generated FEM mesh for each element and the pipeline assembly is presented in Figure 8.

**Figure 9:**
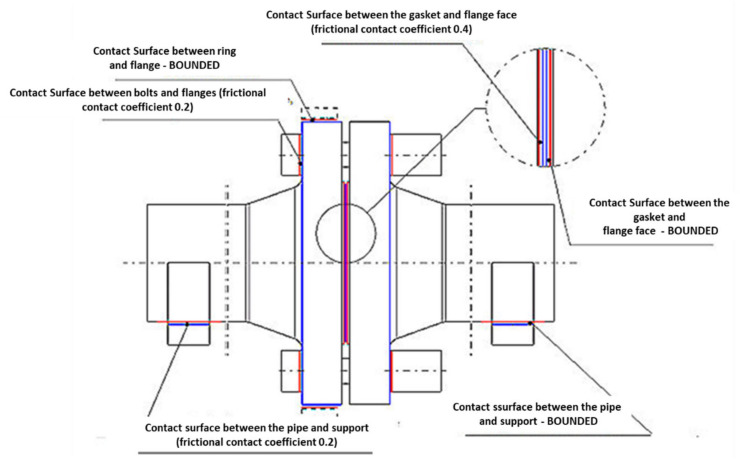
Contact surfaces and kind of contact between the elements of the model.

This model configuration consists of 36,343 finite elements (146,826 nodes). The contact surfaces of the individual elements of the joint were modelled according to Figure 9. The friction was modelled, taking into account the Coulomb model, by introducing the appropriate value of the friction coefficient. “CONTA” and “TARGET” element types were used for discretization of the contact surface. The rest of the contact surface was modelled with “BOUNDED”- type contacts, which prevented mutual displacement of the elements.

The coefficient of friction on the bearing surface of the flanges with the nut and bolt head surface was assumed as 0.15, whereas, in the case of the face contact surface of the gasket with the flange-raised face, the value was assumed as 0.4. The “BOUNDED”-type contact was used on the contact surface of the gasket with the right flange-raised face. The contact ensured bonding of the gasket with the flange, preventing the “escape” of the gasket at the pipeline bending. The types of contact defined between particular elements are presented in Table 3.

**Figure 10:**
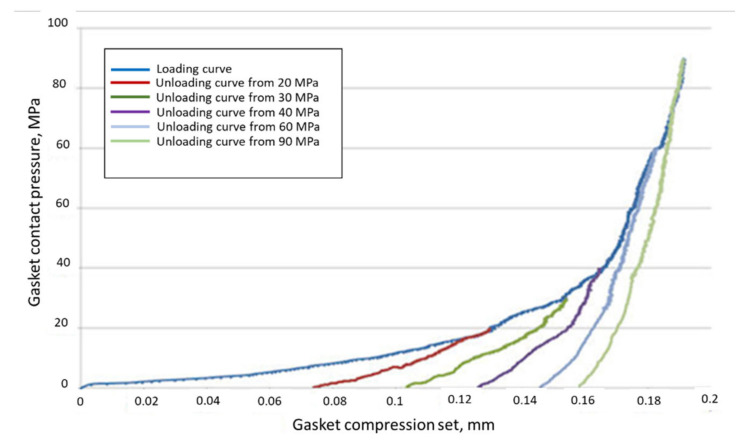
Elastic characteristics of a 2-mm-thick fibre–elastomer gasket.

The material characteristics of steel elements (except the gasket) were modelled as follows: Elasticity modulus *E* = 205 GPa and Poisson’s ratio *v* = 0.3. The gasket’s elasticity characteristics were obtained for the gasket’s tests according to the standards in [13,22]. Sample elastic characteristics of a 2-mm-thick fibre–elastomer gasket are presented in Figure 10.

**Figure 11:**
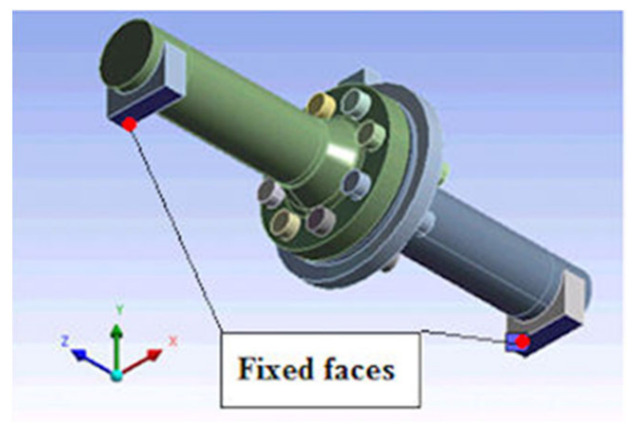
Model fixing surfaces.

Since the test-supporting frame was neglected in the analysis, a fixed support was introduced onto the contact surface of the supports (Figure 11).

**Figure 12:**
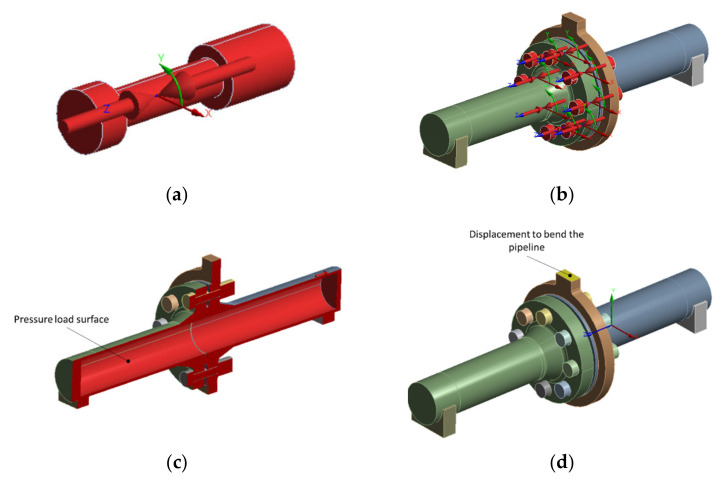
Flange joint load: (**a**) view of a bolt with a marked vector of the bolt-shortening direction; (**b**) view of the bolt assembly in the flanged joint; (**c**) joint load surface with 2 MPa pressure; (**d**) defining the displacement vector towards which the pipeline fragment was bent.

Three kinds of load can be distinguished in the analysed assembly:

1. Preload tension of the bolts (Figure 12a,b). In order to obtain an initial tension force of 52 kN per bolt, it was necessary to enter an appropriate value of the displacement. This value resulted directly from the shortening of the bolt section between the bolt head and the nut. The same approach was employed, e.g., in papers [12,13]. The initial value of the bolt head and nut overlapping the flange-bearing surface, ensuring the bolt-tension value of 52 kN, was 0.32 mm.
2. Internal pipeline pressure of 2 MPa. Figure 12c shows the internal surfaces that the pressure was applied to. In this case, the internal pipeline walls and internal lateral surface of the gasket are the load surfaces.
3. Displacement causing the pipeline bending (Figure 12d). The surface where the displacement vector was defined was the place of the ring connection with a hydraulic exciter in the test stand. A bending simulation was carried out for the following three displacement (pipeline deflection) options: 1.2 mm, 0.8 mm, and 0.4 mm.

**Figure 13:**
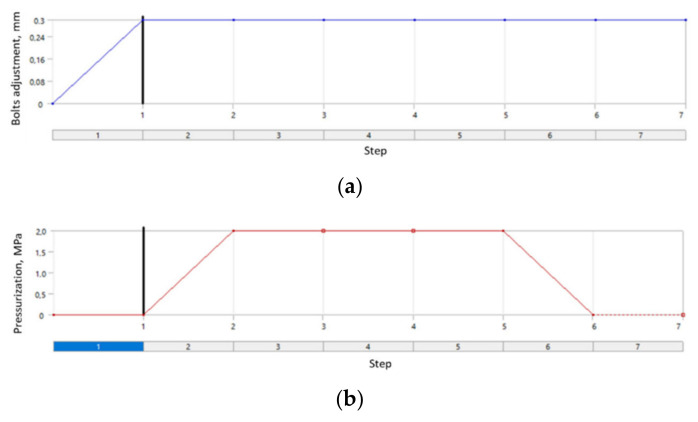

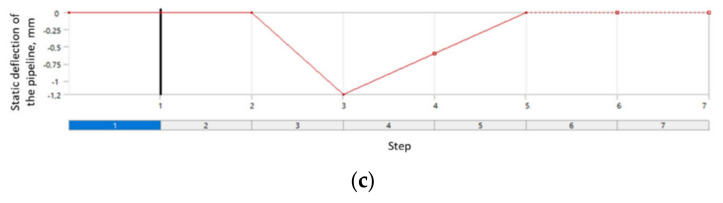
Steps of numerical analysis: (**a**) tensioning of bolts; (**b**) loading with pressure; (**c**) static deflection of the pipeline’s pressurization.

With regard to the fact that the real assembly was subject to loading in steps, the numerical analysis was divided into stages, where each kind of load was applied in the following order: (1) introducing the initial tension of bolts; (2) introducing the static pressure acting on the surfaces of the model; (3) triggering the static deflection of the pipeline to the set displacement value; (4) unloading caused by the displacement; (5) unloading of the system with internal pressure; (6) complete unloading of the bolt tension. The course of each stage of the numerical analysis is presented in Figure 13.

## 3. Results and Discussion

### 3.1. Experimental Test Results

**Figure 14:**
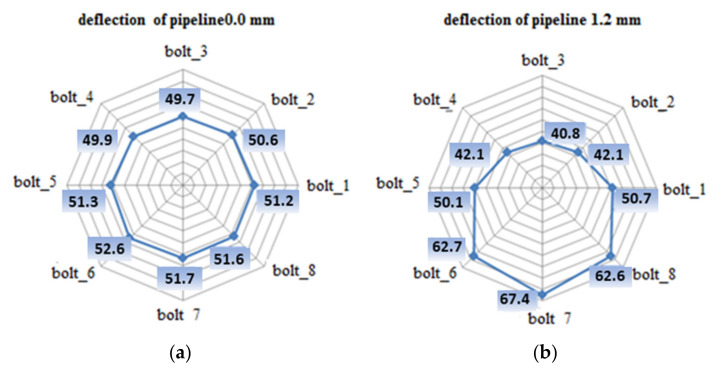
The distribution of the tension force in individual bolts: (**a**) joint before deflection; (**b**) joint deflected by 1.2 mm.

Figure 14 shows the bolt tension values for the case of an unbent joint and a joint subjected to a deflection of 1.2 mm.

**Figure 15:**
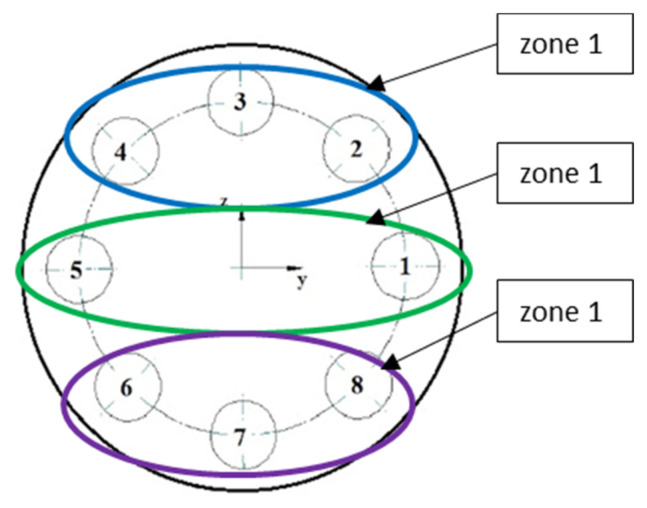
Characteristic bolt-load zones; flange joint subject to static bending (Numbers from 1 to 8 represent the numbers of the bolts.)

As shown in Figure 14b, there is a very large variation in the tension of the bolts in the joint due to deflection. According to the diagram presented in Figure 15, three zones can be distinguished in the bent joint, in which, due to deflection, the bolt tension increases, decreases, or slightly changes. These zones are defined as follows:

- Zone 1, in the area of bolts 2, 3, and 4, where the pipeline deflection and the resulting rotation of the flanges cause unloading of the bolts;
- Zone 2, located at the level of the pipeline-bending axis, where bolts 1 and 5 are located;
- Zone 3, where tension in the bolts increases as a result of deflection, i.e., the location of bolts 6, 7, and 8.

A pipeline deflection value of 1.2 mm causes a decrease in the tension of bolts 2, 3, and 4 to 41.7, 39.4, and 41.7 kN, respectively. For bolts 1 and 5, a minor drop in the load to the value of 51.4 kN per bolt was observed. Tension increases to 63.3, 68.7, and 63.2 kN were recorded for bolts 6, 7, and 8, respectively. Table 4 presents the experiment results for the tension value in the bolts that remained after complete pipeline unloading from the deflection.

**Figure 16:**
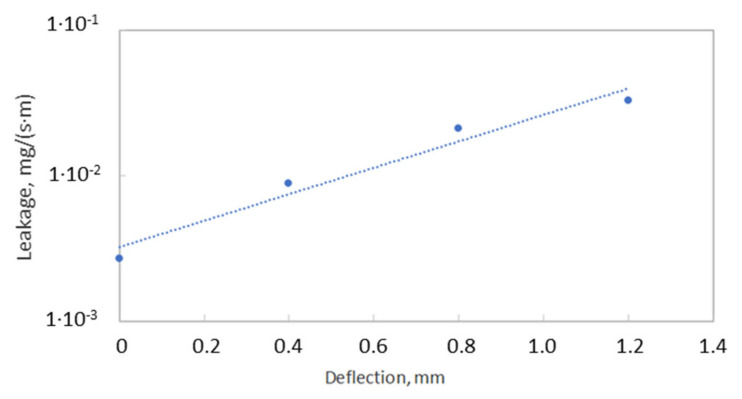
Leakage level vs. pipe deflection.

Figure 16 presents the results of measurements of helium leaking from the joint under the influence of the loading that results from the assembly tension of the bolts, helium pressure, and the deflection of the pipeline. Under the assembly tension of the bolts and the pressure of 20 bar, the leakage value is equal to 2.7 × 10^−3^ mg/(s·m). It is worth noting that this value is within the assumed design tightness class of 0.001 mg/(s·m). It turned out that significant changes in the leakage even occur with small deflections of the pipeline (close to 1 mm). In the case of the deflection by 0.4 mm, the leakage increases to 8.8 × 10^−3^ mg/(s·m). In turn, with the deflection by 0.8 mm, the leakage level increased to 2.1 × 10^−2^ mg/(s·m), while, with the deflection by 1.2 mm, the leakage value was equal to 3.3 × 10^−2^ mg/(s·m), which significantly exceeded the tightness class of 0.001 mg/(s·m). Undoubtedly, the gradual increase in the deflection of the pipeline causes an uneven distribution of pressure on the surface of the gasket, which, in turn, causes the pressure to increase in some areas and decrease in other areas.

The determination of the pressure distribution on the surface of the gasket with regards to the degree of deformation (deflection) of the pipeline was carried out in the next chapter. For this purpose, numerical calculations based on the Finite Element Method were used.

### 3.2. Computation Results and Discussion

**Figure 17:**
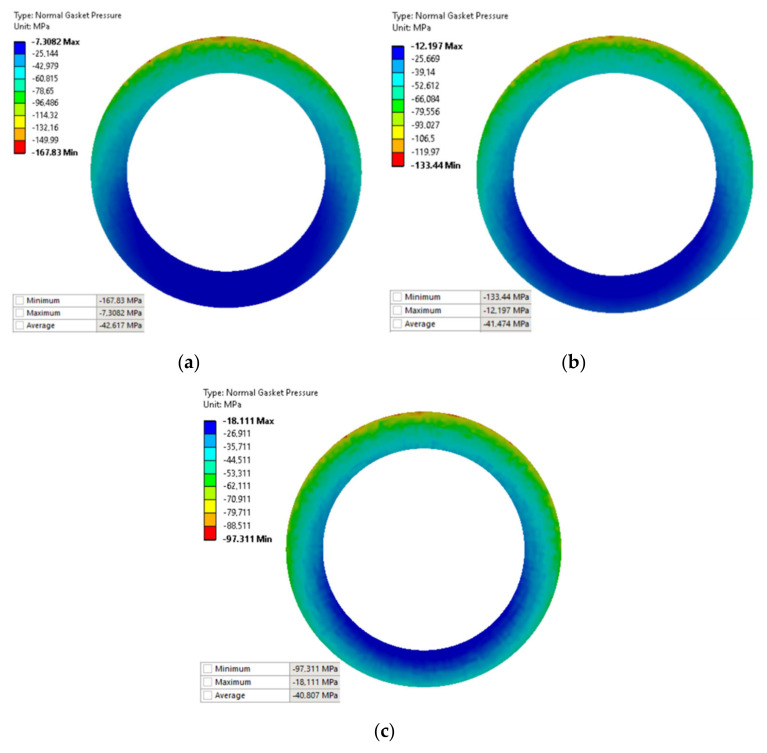
Pressure distribution on the surface of the gasket due to the deflection of the pipeline by: (**a**) 1.2 mm, (**b**) 0.8 mm, and (**c**) 0.4 mm.

The main purpose of the numerical calculations was to assess the contact pressure on the surface of the gasket in the state of deflection of the pipeline. Figure 17 shows maps of pressure distribution on the surface of the gasket for the pipeline deflections of 1.2 mm, 0.8 mm, and 0.4 mm, respectively.

**Figure 18:**
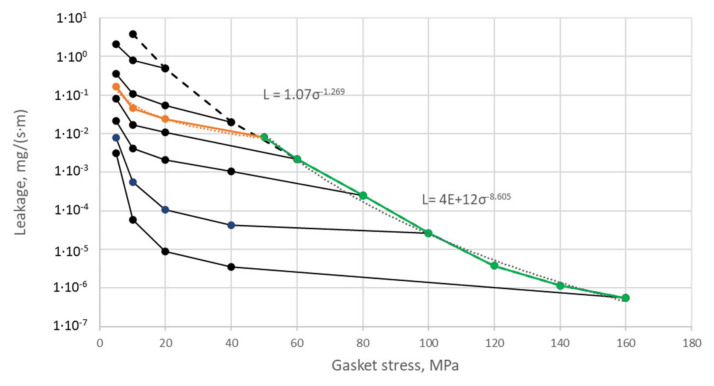
Gasket leakage characteristics.

As can be seen in Figure 17, the gradual increase in the deflection of the pipeline causes an increasingly different pressure in the circumferential direction. The more the deflection of the pipeline increases, the more the pressure on the gasket’s surface located above the axis of the pipeline increases, unlike on the surface located below the axis of bending. In the case of the deflection value of 0.8 mm (see Figure 17b), the minimum and maximum pressure values are 12.2 MPa and 133.4 MPa, respectively. In turn, in the case of the deflection of 1.2 mm (see Figure 17a), the minimum and maximum pressure is equal to 7.3 MPa and 167.8 MPa, respectively. The results of experimental measurements showed that with the deflections of 0.8 mm and 1.2 mm, the leakage from the gasket exceeds the assumed tightness class, within the range of 0.01 mg/(s·m). From the leakage characteristics presented in Figure 18, it can, therefore, be concluded that the average pressure on the gasket’s surface (for 0.8 and 1.2 mm deflections) dropped significantly below 40 MPa.

However, it turns out that an increase in the deflection of the pipeline leads to an increase in the average pressure of the gasket (see average pressures in Figure 17a–c). The mean values of the pressure with the deflections of 0.4 mm, 0.8 mm, and 1.2 mm are 40.8 MPa, 41.5 MPa, and 42.6 MPa, respectively. From the above, it can be concluded that averaging the pressure from the entire gasket’s surface and relating this value to the leakage level is incorrect.

**Figure 19:**
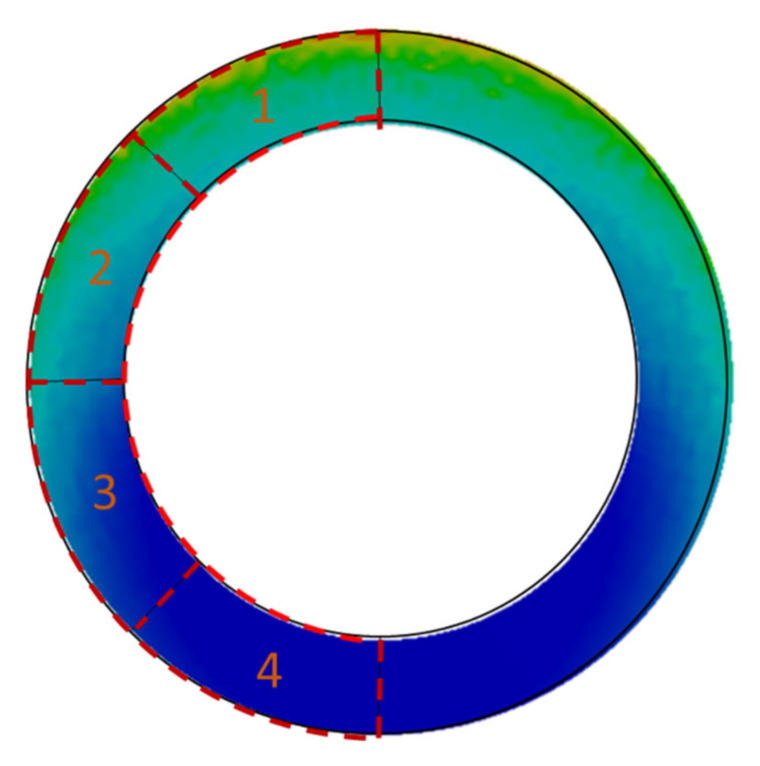
Characteristic zones on the gasket’s surface for calculating the average contact pressure.

For this reason, in the next approach, it was decided to distinguish characteristic gasket zones in which there is an increase or decrease in pressure in relation to the assembly state. Afterwards, the pressures that were calculated in these zones were related to the leakage level. Due to the axial symmetry of the model, four characteristic zones with the same surface were taken into account, as shown in Figure 19.

Average pressures for a given zone, which were calculated in this way, were related to the leakage characteristics presented in Figure 18. Moreover, a criterion was adopted in which it was assumed that if the average pressure in a particular zone is less than or equal to the assembly pressure, the leakage is then calculated according to the following relationship:

(1) $$\mathrm{If}\;\sigma_{i}\leq \sigma_{m}\;\mathrm{then}:\;L=1.0706\;\cdot \;\sigma_{i}^{-1.269}$$

In the second case, i.e., when the average pressure is greater than the assembly pressure, the leakage is calculated according to the following formula:

(2) $$\mathrm{If}\;\sigma_{i}>\sigma_{m}\;\mathrm{then}:\;L=4\times 10^{12}\;\cdot \;\sigma_{i}^{-8.605}$$

The empirical leakage equation presented in Formulas (1) and (2) results directly from the mathematical interpolation of the leakage curve that occurs when the gasket is unloaded and loaded from the assembly pressure of 50 MPa (Figure 18). The leakage value from the entire gasket’s circumference is calculated as the sum of the leakage calculated from individual zones and is related to the average circumference of this zone:

(3) $$L_{c}=\overset{n=4}{\underset{i=1}{\sum }}L_{i}$$

The parameters included in Formulas (1)–(3) are:

*σ_i_—*average contact pressure in particular zone;
*σ_m_—*mean contact pressure obtained at gasket assembly;
*L_i_—*leakage level calculated in the particular zone;
*L_T_—*total leakage level from all zones.

**Figure 20:**
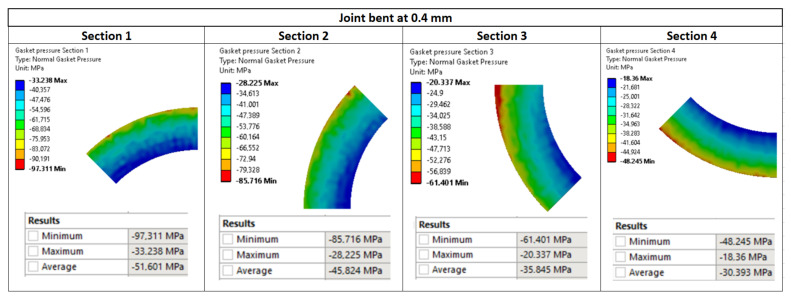
Pressure distribution in the characteristic zones of the gasket—0.4 mm deflection.

**Figure 21:**
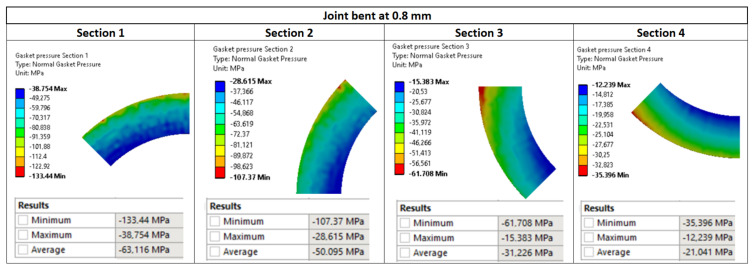
Pressure distribution in the characteristic zones of the gasket—0.8 mm deflection.

**Figure 22:**
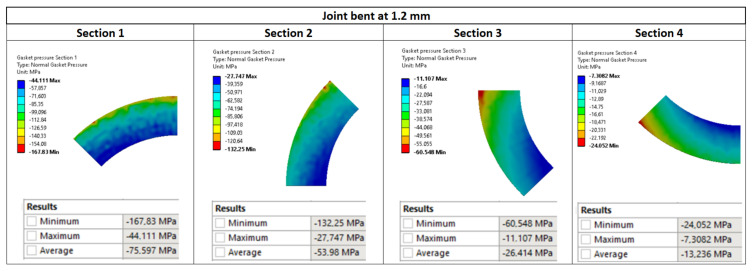
Pressure distribution in the characteristic zones of the gasket—1.2 mm deflection.

Figure 20, Figure 21 and Figure 22 show the pressure values in individual sections with regards to the deflection value. The average values were calculated as a mean value of the nodes located in the particular surface (zone).

The values of leakage calculated using Formula (3) for individual cases of pipeline deflections are presented in Table 5. When analysing these data, it turns out that the averaging of pressure in characteristic zones, and also the proposed method of calculating the leakage, are correct. This is confirmed by small deviations between the values calculated using Formula (3) and those measured experimentally. For the deflection of 0.4 mm, the empirically calculated leakage is equal to 9.86 × 10^−3^ mg/(s·m), and it is close to the experimentally measured value of 8.8 × 10^−3^ mg/(s·m). In the case of the higher deflections of 0.8 mm and 1.2 mm, the leakages calculated using Formula (3) amounted to 1.14 × 10^−2^ mg/(s·m) and 1.56 × 10^−2^ mg/(s·m), respectively, and did not differ much from the values determined experimentally.

**Figure 23:**
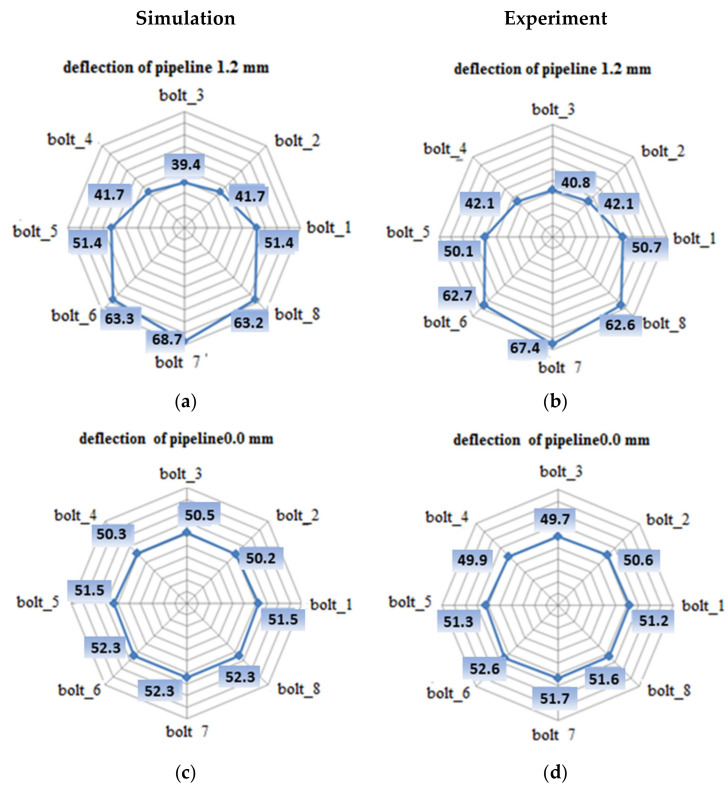
The distribution of the tension in the bolts in the real joint and the calculation model: (**a**) the distribution of bolt tension in the calculation model due to 1.2 mm deflection; (**b**) the distribution of bolt tension in the real joint due to 1.2 mm deflection; (**c**) the distribution of bolt tension in the calculation model before deflection; (**d**) the distribution of bolt tension in the real joint before deflection.

The validation of the numerical model was carried out on the basis of the assessment of the bolt tension under deflection. Figure 23 compares the bolt tension values that were obtained from numerical calculations and experimental measurements for the 1.2 mm deflection. It can be seen that the values obtained numerically do not exceed a 5% error in relation to the experimental data. Therefore, it can be concluded that the adopted boundary conditions of the numerical model accurately reflect the state of the joint’s operation.

## 4. Conclusions

It was demonstrated that pipeline deflection within a minor displacement causes an increase in the diversification of the gasket’s circumferential pressure, and also that it has a significant influence in identifying the actual stress condition the gasket is subject to. Furthermore, it was discovered that the distribution of contact pressure at the pipeline deflection has a significant impact on leakage level. Dividing the gasket area into characteristic sections allowed the leakage level to be calculated based on an empirical formula. Based on the observed changes in tension in the bolts during the experimental and numerical tests, it can be concluded that the assumptions for the numerical model are correct. The numerical model of a flanged-bolted joint constructed based on the acquired assumptions can, therefore, be used as a tool to support the work of constructors, particularly at the stage of analysing the stress and deformation condition of prototypical gaskets.

## Figures and Tables

**Table 1:** Basic geometrical dimensions of a flange joint.

No.	Parameter	Symbol	Value	Unit
Operating parameters				
1.	Pressure	*p*	2	MPa
2.	Fluid temperature	*T*	20	°C
Flanges				
3.	External diameter	*D*	275	mm
4.	Pitch circle diameter	*D_b_*	210	mm
5.	Pipe diameter	*D_p_*	100	mm
6.	Flange thickness	*t_f_*	38.5	mm
7.	Material	*-*	1.4301	-
Pipe				
8.	External diameter	*D_pe_*	114.2	mm
9.	Wall thickness	*t_s_*	2.6	mm
10.	Material	*-*	1.4301	-
Gasket				
11.	Outer diameter	*d_o_*	152	mm
12.	Inner diameter	*d_i_*	112	mm
13.	Gasket thickness	*t_g_*	1 and 2	mm
Bolts				
14.	Number of bolts	*n_b_*	8	-
15.	Bolt strength	*b_s_*	12.9	-
16.	Bolt diameter	*M*	24	mm

**Table 2:** Results of the flexibility analysis.

*L* (mm)	*σ*_1_ (MPa)	*δ* (mm)
1850	31.8	0.4
2250	35.86	0.8
2500	38.76	1.2

**Table 3:** Types of contact for individual interacting elements of the model.

Elements in Contact	Kind of Contact	Contact Elements
Gasket/Left flange	“Frictional”	CONTA/TARGET
Gasket/Right flange	“Bounded”	–
Ring/Left flange	“Bounded”	–
Support/Left flange	“Frictional”	CONTA/TARGET
Support/Right flange	“Bounded”	–
Bolts/Flanges	“Frictional”	CONTA/TARGET

**Table 4:** Bolts’ tensions as a function of pipeline deflection.

Bolt’s Tension		Number of Bolts							
		1	2	3	4	5	6	7	8
Deformation		kN	kN	kN	kN	kN	kN	kN	kN
1.2	mm	50.7	42.1	40.8	42.1	50.1	62.7	67.4	62.6
0.8	mm	50.5	48.4	46.2	48.7	50.6	54.9	58.3	54.7
0.4	mm	50.2	50.6	50.8	51.3	50.3	52.0	52.7	51.8

**Table 5:** Summary of calculated leakage values.

Deflection 1.2 mm			Deflection 0.8 mm			Deflection 0.4 mm		
Section	Stress	Leakage	Section	Stress	Leakage	Section	Stress	Leakage
–	MPa	mg/(s·m)	–	MPa	mg/(s·m)	–	MPa	mg/(s·m)
1.	75.6	6.84 × 10^−5^	1.	63.12	0.000323	1.	47.03	0.00202
2.	54	0.001238	2.	50.9	0.002059	2.	43.89	0.002205
3.	26.4	0.004203	3.	31.23	0.003396	3.	37.67	0.002677
4.	13.23	0.0101	4.	21.04	0.005606	4.	34.84	0.002956
Total	1.56 × 10^−2^ mg/(s·m)		Total	1.14 × 10^−2^ mg/(s·m)		Total	9.86 × 10^−3^ mg/(s·m)	

## Data Availability

Data available upon request from the corresponding author.
